# A54T polymorphism in the fatty acid binding protein 2 studies in a Saudi population with type 2 diabetes mellitus

**DOI:** 10.1186/1476-511X-13-61

**Published:** 2014-04-01

**Authors:** Khalid Khalaf Alharbi, Imran Ali Khan, Mohammad D Bazzi, Nasser M Al-Daghri, Tarique N Hasan, May Salem Alnbaheen, Fawiziah Khalaf Alharbi, Yazeed A Al-Sheikh, Rabbani Syed, Mourad AM Aboul-Soud

**Affiliations:** 1 Department of Clinical Laboratory Sciences, College of Applied Medical Sciences, King Saud University, P.O. Box 10219, Riyadh 11433, Kingdom of Saudi Arabia; 2 Department of Biochemistry, College of Science, King Saud University, Riyadh 11451, Kingdom of Saudi Arabia; 3 Biomarkers research programme, Department of Biochemistry, College of Science, King Saud University, Riyadh 11451, Kingdom of Saudi Arabia; 4 Department of Food Science and Nutrition, College of Food and Agricultural Sciences, King Saud University, Riyadh 11451, Kingdom of Saudi Arabia; 5 Stem Cell unit, Department of Anatomy, College of Medicine, King Khalid University Hospital, Riyadh 11461, Kingdom of Saudi Arabia; 6 Prepratory Year, Saudi Electronic University, Riyadh, Kingdom of Saudi Arabia; 7 Department of Biology Science, College of Science and Arts, Al-Qassim University, P.O. Box 1300, Buraidah 51431, Kingdom of Saudi Arabia

**Keywords:** A54T polymorphism, FABP2 gene, Type 2 diabetes mellitus, Saudi population

## Abstract

**Background:**

Fatty acid-binding protein 2 (*FABP2*) is an intracellular protein expressed exclusively in the enterocytes of proximal small intestine. *FABP2* has a high affinity for saturated and unsaturated long-chain fatty acids and is believed to be involved in the absorption and transport of dietary fatty acids.

**Methods:**

This is a case–control study conceded in 438 T2DM cases and 460 subjects with normal glucose levels and non-obese considered as healthy controls. Allelic discrimination was performed using TaqMan single-nucleotide polymorphism was carried out by real time-polymerase chain reaction (RT-PCR) assays using purified DNA.

**Results:**

Clinical data and anthropometric measurements except age, glucose levels and lipid profile of the patients were significantly different from those of the controls (*p <* 0.05). Statistical analyses failed to show any type of significant association of the polymorphism between cases and controls. However logistic regression analyses was suggests that the TT genotype is significantly associated with male patients (*p =* 0.001). None of the allele or genotypes of *FABP2* A54T was associated with T2DM cases versus the controls (AT genotype, OR = 0.85 (0.64-1.12), *p* = 0.25; TT genotype, OR = 0.66 (0.39-1.11), *p =* 0.11; T allele, 0.82 (0.67-1.02), *p* = 0.08).

**Conclusion:**

In conclusion, this study suggests that the above named variant in *FABP2* gene is not potential contributor to the risk of T2DM and related traits in a Saudi population. However TT genotype is a risk factor for the disease in males.

## Introduction

Type 2 diabetes mellitus (T2DM) is a chronic degenerative disease, phenotypically and genetically characterized by insulin resistance (IR) in insulin-target tissues, and impaired insulin secretion from pancreatic β-cells [1]. T2DM affects nearly 31.6% individuals in the Kingdom of Saudi Arabia [2]. The interaction of genetic and environmental factors is universally acknowledged as the primary underlying T2DM mechanism. The T2DM risk in the first degree relatives of T2DM patients is 3.62 times that in the common population, so the researchers of various countries make great efforts to explore the T2DM susceptible genes. Once the T2DM susceptible genes are sought out, it means that the T2DM prevention clues have been found. It is an effective measure to screen the T2DM susceptible population and prevent T2DM progress. It is now generally considered that T2DM is not a sole disorder, but a multigenic disorder with extensive heredity heterogeneous, categorized by high levels of glucose and metabolic disorders [3,4]. Obesity is one of the complication for T2DM and the leading cause of preventable deaths and a serious health complications in the Saudi Arabia [5].

Genome Wide Association Studies (GWAS) are powerful tools to identify genetic variants that are associated with common diseases. So far, GWAS identified at least 50–52 genetic loci robustly associated with T2DM [6]. Several genetic markers have now been implicated for T2DM development pathways involved in the disease [7,8]. Fatty acid binding protein 2 (*FABP2*) has been studied as one of the marker, due to its role in the uptake, intracellular metabolism and excretion of long chain fatty acids. This gene is located on 4q28-q31 chromosomal region, consist of approximately 3.4 kbs. Many polymorphisms in *FABP2* gene have already linked with metabolic phenotypes. The most extensively studies polymorphism was Alanine54threnonine (A54T) substitution at codon 54 of exon 2 that results from G to A nucleotide substitution and affects primary protein structure [9,10]. This change affects the ability of the protein to transport dietary fatty acids, which may elevate saturated fatty acids level in the serum which might induce endothelial dysfunction leading to increased cardiovascular mortality [11].

The aim of the present study was to investigate the contribution of A54T genetic polymorphism identified by Baier et al [12] in the *FABP2* variants to risk of T2DM in a Saudi population, since this polymorphism results in a functionally altered *FABP2* protein which confers susceptibility to metabolic disorders like T2DM, thereby to contribute to the personalized prevention of this condition and A54T polymorphism has been associated with T2DM disease in many but not all studies.

## Materials and methods

### Selection of subjects

This is a case–control study carried out in King Saud University, Riyadh, Saudi Arabia. In this study we have collected in about 900 subjects; 438 T2DM cases and 460 healthy controls from the capital city. All the normal controls were selected from the general population based on normal glucose values and non-obese subjects. The details of the collection of patients, inclusion and exclusion criteria were described in the prior publication [1]. Ethical approval for the study was achieved from the ethics committee, King Saud University. Written informed consent was obtained from each patient. Anthropometric and biochemical measurements of the patients involved in this study has been described in the prior publication [1].

#### *DNA extraction and genotyping*

5 mL of the venous blood was collected into plain (coagulated) and EDTA (anticoaggulated) tubes. Plain vacutainer consist of 3 mL of the serum sample was used to analyze the biochemical parameters and 2 mL of the EDTA sample was used for molecular analysis. Genomic DNA was extracted from peripheral blood leukocytes using Norgen DNA extraction kit (Norgen Biotek corp, Canada). DNA samples were stored at -80°C. The rs1799883 polymorphism was genotyped using a TaqMan® SNP genotyping assay (Assay ID: C_2834835_10) on a 7300HT sequence detection system (Applied Biosystems, USA). Primers and probes were obtained from Applied Biosystems as Assays-by-Design™. Cases and controls were ensured to have even treatment during the assay procedure, and each plate included negative controls (with no DNA). Plates were read on the ABI Prism 7300 using the Sequence Detection Software (Applied Biosystems) using 40 PCR cycles (92°C denaturation for 15 seconds, 60°C annealing/extension for 60 seconds). Measurements were repeated for samples with failed genotypes. Assays that did not show >95% concordance were discarded and replaced with alternative assays with the same tagging properties.

### Statistical analysis

All statistical analysis was carried out using the statistical program i.e. statistical package for social sciences (IBM SPSS 19.0 SPSS Inc., Chicago, USA). The difference of mean age of patients and controls were significantly different (Table 1). Hence in order to overcome the significance difference of mean age, logistic regression was performed and it was found that patients with ages 35 (1 patient), 72 (2 patients), 77 (2 patients), 84 (2 patients), 85 (2 patients) and 86 (2 patients) years were redundant whereas rest all the patient ages were significantly not different from those of controls. Hence those patients were excluded from the further calculations if genotypic and allelic analyses. The sex ratio of patients and controls were also different significantly. So, separate multiple analyses after logistic regressions were performed for patient and controls. The allele and genotype frequencies of *FABP2* gene in patients were compared to controls by chi-square analysis. The distribution of the genotypes deviates from Hardy-Weinberg equilibrium (HWE). Clinical characteristics of all the subjects were expressed as mean ± SD. Continuous variables were compared between the groups using two-tailed student *t*-test. Odds ratios (ORs) and 95% CI, with adjustment for age, sex and BMI were calculated using multiple logistic regression analysis. All tests were conducted at the *p <* 0.05 level of significance.

**Table 1:** Demographic characteristics of the study population

	**Controls (*n =*** **460)**	**T2DM (*n =*** **438)**	***p***
Age (Years)	45.99 ± 7.77	53.5 ± 10.78	<0.0001
Weight (kg)	76.61 ± 14.52	77.37 ± 13.55	0.41
Height (cm)	161.25 ± 8.79	161.10 ± 9.30	0.80
Body mass index (kg/m^2^)	29.22 ± 5.58	29.9 ± 5.89	0.95
Sex: Male/Female	(52.6%)/(47.4%)	(56.8%)/(43.2%)	0.0002
SBP (mmHg)	114.80 ± 8.04	125.83 ± 9.96	<0.0001
DBP (mmHg)	75.81 ± 6.20	81.25 ± 4.82	0.0001
Waist (cms)	89.75 ± 14.19	95.3 ± 18.96	<0.0001
Hips (cms)	101.75 ± 14.72	99.64 ± 16.52	0.61
FBS (mmol/L)	5.23 ± 0.61	12.92 ± 4.60	<0.0001
Triglycerides (mmol/L)	1.62 ± 0.86	2.24 ± 1.62	<0.0001
Cholesterol (mmol/L )	5.04 ± 0.96	5.61 ± 1.26	<0.0001
HDL-C (mmol/L )	0.64 ± 0.23	0.84 ± 0.37	<0.0001
LDL-C (mmol/L)	3.66 ± 0.85	3.76 ± 1.05	0.0008
Glucose (mmol/L)	5.7 ± 1.2	9.4 ± 1.5	0.0002
Insulin (μU/mL)	12.5 ± 1.8	16.2 ± 2.2	0.0002
HOMA-IR	3.15 ± 1.9	6.8 ± 2.4	0.00008

Note: Data represented as mean ± SD for continuous variables, pValues for independent *t*-test are given. A *p*-value significant at <0.05. *NA*, Not Analyzed/Not Applicable.

## Results

### Clinical characteristics

In this case–control study, we have carried out in almost 900 subjects; 438 patients with T2DM (216 females and 244 males, 45.99±7.77 years old) and 460 normal control subjects (200 female and 238 male, 53.5 ± 10.78 years old). The mean age was 60 years for patients and 59 years for the control group. Clinical and anthropometric data are revealed in Table 1 for T2DM patients and control subjects. The results show that T2DM subjects were significantly older than controls but anthropometric measurements and hip were not significant (*p >* 0.05). T2DM subjects appear to have higher levels of SBP, DBP, fasting glucose, insulin, HOMA-IR and lipid profile and waist (*p <* 0.05).

### Genotype frequencies

Results for the genotypic distribution of *FABP2* A54T polymorphism and the frequency of A and T alleles in patients and controls have been tabulated in Table 2. The genotype distribution for A54T polymorphism showed no deviation from HWE in both the case and control groups (*χ*^*2*^*=* 0.37, *p =* 0.54). Genotyping of the A54T polymorphism in the *FABP2* gene revealed that the allelic frequency of T allele was 0.285 in cases and 0.248 in controls (OR-0.82 (95% CI = 0.67-1.02); *p =* 0.08). There were no significant differences in both the genotypic and allelic frequencies between the cases as well as controls. However upon multiple logistic regression analysis TT genotype was found to be associated with male patients (OR = 1.24 95% CI = 0.09-0.60, *p =* 0.001) (Tables 3 and 4). *FABP2* genotypic frequencies of A54A, A54T, and T54T were 56.5%, 37.2%, and 6.3% in the control group, 51.52%, 39.81% and 8.66% in the T2DM group; allelic frequencies of Ala and Thr were 0.75 and 0.25 for the control group, 0.71 and 0.29 for the T2DM group. The odds ratio for any genotype of A54T SNP was not significantly related with the risk of developing T2DM in this studied population (for AA + AT vs TT; OR-0.7 (95% CI = 0.42-1.17); *p =* 0.12 and for TT + AT vs AA; OR-1.22 (95% CI = 0.93-1.59); *p =* 0.25) from the Saudi population.

**Table 2:** **Distribution of *FABP2 *** **A54T genotypes and alleles of this study**

** Genotype**	**Patients’ *n *** **(%)**	**Controls *n *** **(%)**	**OR (CI = 95%)**	***P *** **value**	** χ** ***2***
					**1 d. f.**
**AA**	220 (51.52)	260 (56.52)	1 (reference)	1 (reference)	
**AT**	170 (39.81)	171 (37.17)	0.85 (0.64-1.12)	0.25	1.12
**TT**	37 (8.66)	29 (6.30)	0.66 ( 0.39-1.11)	0.11	2.43
**A**	610 (71.42)	691 (75.10)	1 (reference)	1 (reference)	
**T**	224 (28.57)	229 (24.89)	0.82 (0.67-1.02)	0.08	3.06
**AA + AT vs TT**			0.7 (0.42-1.17)	0.18	1.79
**TT + AT vs AA**			1.22 (0.93-1.59)	0.13	2.22

The genotypic and allelic frequencies and dominant as recessive models of patients are significantly not different from controls.

**Table 3:** Genotypic distribution according to sex

**Patients**			
	**AA**	**AT**	**TT**
Male	122	95	31
Female	98	86	6
Total	220	181	37
Controls			
Male	136	95	13
Female	124	76	16
Total	260	171	29

**Table 4:** Calculation for the association of A54T genotypes with T2DM patients and control in male and female sexes

			**OR**	**95% CI**	***P *** **Value**
Patients					
	Genotypes				
	AT				
		Male	1 (reference)		
		Female	1.1	(0.74-1.65)	0.62
	TT				
		Male	1 (reference)		
		Female	1.24	(0.09-0.60)	0.001
Controls					
	Genotypes				
	AT				
		Male	1 (reference)		
		Female	0.87	(0.59-1.29)	0.5
	TT				
		Male			
		Female	1.35	(0.62-2.91)	0.44

*OR*, Odds ratio, 95% CI-95% confidence interval; multiple logistic regression calculated by SPSS v.19, AA genotype was set as zero because redundancy

## Discussion

*FABP2* is an intracellular protein expressed in the villus tips of the small intestine, has a high affinity for saturated and unsaturated long-chain fatty acids and is believed to be involved in the absorption and transport of dietary fatty acids [13]. The association between fatty acid metabolism and IR is well known, and the *FABP2* gene has been suggested as a possible candidate gene in the development of IR and T2DM [14]. A54T polymorphism in *FABP2* was investigated as a possible genetic factor associated with T2DM and Obesity. Studies examining the association of A54T polymorphism with IR, T2DM and Obesity are contradictory and inconclusive [10]. The product of T allele of *FABP2* possesses a greater affinity for long-chain FA than the A allele [15]. In addition, individuals with the T allele of this polymorphism were more insulin resistant than were those with the A allele [12]. The T allele was also shown to be associated with higher plasma levels of LDL-C [16] and dyslipidemia (high plasma concentration of triglycerides and low concentration of HDL-C) [17]. In addition, the T allele of A54T polymorphism has previously been associated with atherothrombotic cerebral infarction in individuals with metabolic syndrome [18] and a parental history of stroke in the Swedish population [19]. Moreover, it was associated with a 2- to 3.5- fold increase in cardiovascular risk in dyslipidemic men with diabetes compared with their dyslipidemic nondiabetic counterparts [20]. We have now shown that A54T polymorphism was not significantly associated with T2DM, with the minor allele representing the risk factor for this condition.

In the present study we investigated the association of *FABP2* A54T polymorphism with T2DM in a Saudi population. To the best of our knowledge, this is the first study investigating the association of A54T polymorphism in a Saudi population. The frequency of T allele of A54T polymorphism was 0.29 similar to Greek and Caucasian population [21-23]. A54T polymorphism was identified via linkage disequilibrium map, as a haploblock spanning 50 kb that includes 22 SNPs. However, the T54 allele is present in only one of six possibilities among the frequent haplotypes (>2%). It is interesting that in this haploblock, there are no other known or putative genes except for *FABP2*[24].

Previous studies of A54T polymorphisms investigated lipid related diseases such as coronary artery disease (CAD) [25]. Canani et al [26] showed that A54T polymorphism confers susceptibility to renal disease in T2DM patients. There are very few reports on the association of this polymorphism with T2DM. Several studies from non-European ethnic backgrounds have reported a positive association between *FABP2* variants and T2DM. A54T polymorphism was carried out in relation to multiple diseases and the reports are summarize in Table 5, which shows positive association with T2DM and a combination of other diseases like chronic kidney disease, Microalbminuria, postprandial fatty acids and with the glyburide therapy. In our study, we have examined only T2DM (*n =* 438) samples and that have not examined its association with other disease.

**Table 5:** Association Studies of FABP2 A54T gene polymorphism on different ethnic groups

**S. No**	**Population**	**Cases**	**Controls**	**Association**	**Disease**
1	Germany	-	68	No	Obesity
2	Japan	228	813	Yes	MI + chronic kidney disease
3	Japan	636	1106	Yes	Chronic kidney disease + T2DM
4	Japan	313	971	Yes	Atherothrombic cerebral infarction
5	Brazil	72	37	Yes	T2DM + microalbminuria
6	Mexico	-	131	No	Lipid metabolism
7	Brazil	513	529	Yes	Renal disease + T2DM
8	Brazil	26	529	Yes	Postprandial fatty acids + T2DM
9	Spain	108	101	Yes	Hypercholesterolemic
**10**	**Present Study**	**438**	**460**	**No**	**T2DM**

There are several studies denote no association of the T allele with T2DM [27-29] and our study is supporting the above mentioned studies. In some studies, subjects were distributed in homozygous for the A allele (AA) and T allele carriers (AT and TT), mainly because there were only few subjects homozygous for the variant [23,30]. We observed the association of A54T polymorphism with T2DM according to all possible genetic models (i.e. additive, dominant and recessive). No association of A54T polymorphism was found with T2DM according to any genetic model used, a finding shared by other studies that examined such an association [31]. There are limitations to the study design as it is a case–control observational study rather than cross sectional or prospective study. We have chosen only one SNP (A54T) to study the T2DM disease.

In conclusion, the present study provides no evidence of any association between A54T polymorphism (rs1799883) in *FABP2* gene and T2DM, suggesting that A54T polymorphism is not a major risk factor for the T2DM. Overall, this study indicates that A54T polymorphism could not affect directly the presence of T2DM due to the differential absorption of long chain fatty acids in the presence of the T allele. This is the first study finding the interaction between *FABP2* and T2DM in Saudi population. Further functional studies as well as well-characterized larger molecular epidemiological studies are necessary to confirm our findings.

## Competing interest

The authors declare that they have no competing interests.

## Authors’ contributions

AKK and BMD were main investigator of this study. IAK has plan the study protocol, drafted the manuscript, prepared the final version of the manuscript and CA. ANM has confirmed the T2DM samples. HTN has helped in the revision of the manuscript. AMS and AFK helped with the genotyping and interpretation of the data. SR, took care about the sample collection. AYA and AMAM has participated in the study coordination and took part in the statistical analysis. All authors read and approved the final manuscript.

## References

[B1] Alharbi KK Khan IA Munshi A Alharbi FK Al-Sheikh Y Al-Nbaheen MS Association of the genetic variants of insulin receptor substrate 1 (IRS-1) with type 2 diabetes mellitus in a Saudi population Endocrine 2014 [Epub ahead of print] 10.1007/s12020-014-0177-2 24493031

[B2] Al-Attas O Al-Daghri NM Alokail M Abd-Airahman S Vinodson B Sabico S Metabolic benefits of Six-month Thiamine supplementation in patients with and without type 2 diabetes mellitus Clin Med Insights Endocrinol Diab 2014 7 1 6 10.4137/CMED.S13573 PMC3921172 24550684

[B3] Li YY Wang LS Lu XZ Yang ZJ Wang XM Zhou CW Xu J Qian Y Chen AL CDKAL1 gene rs7756992 A/G polymorphism and type 2 diabetes mellitus: a meta-analysis of 62, 567 subjects Sci Rep 2013 4 3 3131 24185407 10.1038/srep03131 PMC3816287

[B4] Shen HB Shen DB An epidemiological study on genetic agent in type 2 diabetes mellitus China Public Health 1999 15 492 494

[B5] Al-Daghri NM Alkharfy KM Al-Attas OS Krishnaswamy S Mohammed AK Albagha OM Alenad AM Chrousos GP Alokail MS Association between type 2 diabetes mellitus-related SNP variants and obesity trait in a Saudi population Mol Bio Rep 2014 41 1731 1740 10.1007/s11033-014-3022-z 24435973

[B6] Wilson PW Meigs JB Sullivan X Fox CS Nathan DM D’Agostino RB prediction of incident diabetes mellitus in middle-aged adults: the Framingham offspring study Arch Intern Med 2007 167 1068 1074 10.1001/archinte.167.10.1068 17533210

[B7] Albuquerque D Nobrega C Manco L Association of FTO polymorphisms with obesity and obesity-related outcomes in Portuguese children PLoS One 2013 8 1 e54370 10.1371/journal.pone.0054370 23342142 PMC3544709

[B8] Perry JRB Vought BF Yengo L Amin N Dupuis J Ganser M Grallert H Navarro P Li M Qi L Steinthorsdottir V Scott RA Almgren P Arking DE Aulchenko Y Balkau B Benediktsson R Bergman RN Boerwinkle E Bonnycastle L Burtt NP Campbell H Charpentier G Collins FS Gieger C Green T Hadjadj S Hattersley AT Herder C Hofman A Stratifying type 2 diabetes Cases by BMI identifies genetic risk variants in LAMA1 and enrichment for risk variants in lean compared to obese cases PLoS Genet 2012 8 5 e1002741 10.1371/journal.pgen.1002741 22693455 PMC3364960

[B9] Mansego ML Martinez F Martinez-Larrad MT Zabena C Rojo G Morcillio S Soriquer F Martin-Escudero JC Serrano-Rios M Redon J Chaves FJ Common variants of the liver fatty acid binding protein gene influences the risk of type 2 diabetes and insulin resistance in Spanish population PLoS One 2012 7 3 e31853 10.1371/journal.pone.0031853 22396741 PMC3292554

[B10] Tavridou A Arvanitidis KI Tiptiri-Kourpeti A Petridis I Ragia G Kyroglou S Christakidis D Manolopoulos VG Tha 54 allele of fatty acid binding protein 2 gene is associated with obesity but not type 2 diabetes mellitus in a Caucasian population Diabetes Res Clin Pract 2009 84 132 137 10.1016/j.diabres.2009.02.022 19324445

[B11] Freathy RM Mook-Kanamori DO Sovio U Prokopenko I Timpson NJ Berry DJ Warrington NM Widen E Hottenga JJ Kaakinen M Lange LA Bradfield JP Kerkhof M Marsh JA Magi R Chen CM Lyon HN Kirin M Adair LS Aulchenko YS Bennett AJ Borja JB Bouatia-Naji N Charoen P Coin LJ Cousminer DL de Geus EJ Deloukas P Elliott P McCarthy MI Early Growth Genetics (EGG) Consortium Variants in ADCY5 and near CCNL1 are associated with fetal growth and birth weight Nat Genet 2010 42 430 435 10.1038/ng.567 20372150 PMC2862164

[B12] Baier LJ Sacchettini JC Knowler WC Eads J Paolisso G Tataranni PA Mochizuki H Bennett PH Bogardus C Prochazaka M An amino acid substitution in the human intestinal fatty acid binding protein is associated with increased fatty acid binding, increased fat oxidation, and insulin resistance J Clin Invest 1995 95 1281 1287 10.1172/JCI117778 7883976 PMC441467

[B13] Albala C Villaroel A Santos JL Angel B Lera L Liberman C Sanchez H Perez-Bravo F FABPS Ala54Thr polymorphism and diabetes in Chilean elders Diabetes Res Clin Pract 2007 77 245 250 10.1016/j.diabres.2006.12.006 17292994

[B14] Zhao T Zhao J Yang W Association of the fatty acid-binding protein 2 gene Ala54Thr polymorphism with insulin related and blood glucose: a meta-analysis in 13451 subjects Diabetes Metab Res Rev 2010 26 357 364 10.1002/dmrr.1085 20578207

[B15] Lowe JB Sacchettini JC Laposata M McQuillan JJ Gordon JI Expression of rat intestinal fatty acid-binding protein in Escherichia coli: purification and comparison of ligand binding characteristics with that of Escherichia coli-derived rat liver fatty acid-binding protein J Biol Chem 1987 262 5931 5937 3553183

[B16] Yamada K Yuan X Ishiyama S Koyama K Ichikawa F Koyanagi A Koyama W Nonaka K Association between Ala54Thr substitution of the fatty acid-binding protein 2 gene with insulin resistance and intra-abdominal fat thickness in Japanese men Diabetologia 1997 40 706 710 10.1007/s001250050737 9222651

[B17] Galluzzi JR Cupples LA Otvos JD Wilson PW Schaefer EJ Ordovas JM Association of the A/T54 polymorphism in the intestinal fatty acid binding protein with variations in plasma lipids in the Framingham Offspring Study Atherosclerosis 2001 159 417 424 10.1016/S0021-9150(01)00517-2 11730822

[B18] Guettier JM Georgopoulos A Tsai MY Radha V Shanthirani S Deepa R Gross M Rao G Mohan V Polymorphisms in the fatty acid-binding protein 2 and apolipoprotein C-III genes are associated with the metabolic syndrome and dyslipidemia in a South Indian population J Clin Endocrinol Metab 2005 90 1705 1711 10.1210/jc.2004-1338 15598690

[B19] Yamada Y Kato K Oguri M Yoshida T Yokoi K Watanabe S Metoki N Yoshida H Satoh K Ichihara S Aoyagi Y Yasunaga A Park H Tanaka M Nozawa Y Association of genetic variants with atherothrombotic cerebral infarction in Japanese individuals with metabolic syndrome Int J Mol Med 2008 21 801 188 18506375

[B20] Carlsson M Orho-Melander M Hedenbro J Almgren P Groop LC The T 54 allele of the intestinal fatty acid-binding protein 2 is associated with a parental history of stroke J Clin Endocrinol Metab 2000 85 2801 2804 10946885 10.1210/jcem.85.8.6751

[B21] Sipilainen R Uusitupa M Heikkinen S Rissanen A Laakso M Variants in the human intestinal fatty acid binding protein 2 gene in obese subjects J Clin Endocrinol Metab 1997 82 2629 2632 10.1210/jcem.82.8.4179 9253345

[B22] Fisher E Li Y Burwinkel B Kuhr V Hoffman K Mohlig M Spranqer J Pfeiffer A Boeing H Schrezenmeir J Doring F Preliminary evidence of FABP2 A54T polymorphism associated with reduced risk of type 2 diabetes and obesity in women from a German cohort Horm Metab Res 2006 38 341 345 10.1055/s-2006-925400 16718632

[B23] Pihlajamaki J Rissanen J Heikkinen S Karjalainen L Laakso M Codon 54 polymorphism of the human intestinal fatty acid binding protein 2 gene is associated with dyslipidemias but not with insulin resistance in patients with familial combined hyperlipidemia Arterioscler Thromb Vasc Biol 1997 17 1039 1044 10.1161/01.ATV.17.6.1039 9194752

[B24] Tuomi T Carlsson A Li H Isomaa B Miettinen A Nilsson A Nissen M Ehrnstrom BO Forsen B Snickars B Lahti K Forsblom C Saloranta C Taskinen MR Groop LC Clinical and genetic characteristics of type 2 diabetes with and without GAD antibodies Diabetes 1999 48 150 157 10.2337/diabetes.48.1.150 9892237

[B25] Ohrvall M Berglund L Salminen I Lithell H Aro A Vessby B The serum cholesterol ester fatty acid composition but not the serum concentration of alpha tocopherol predicts the development of myocardial infarction in 50-year-old men: 19 years follow-up Atherosclerosis 1996 127 65 71 10.1016/S0021-9150(96)05936-9 9006806

[B26] Canani LH Capp C Ng DP Choo SG Maia AL Nabinger GB Santos K Crispim D Roisemberg I Krolewski AS Gross JL The fatty acid-binding protein-2 A54T polymorphism is associated with renal disease in patients with type 2 diabetes Diabetes 2005 54 3326 3330 10.2337/diabetes.54.11.3326 16249461

[B27] Lei HH Coresh J Shuldiner AR Boerwinkle E Brancati FL Variants of the insulin receptor substrate-1 and fatty acid binding protein 2 genes and the risk of type 2 diabetes, obesity, and hyperinsulinemia in African-Americans: the Atherosclerosis Risk in Communities Study Diabetes 1999 48 1868 1872 10.2337/diabetes.48.9.1868 10480621

[B28] Ito K Nakatani K Fujii M Katsuki A Tsuchihashi K Murala K Goto H Yano Y Gabazza EC Sumida Y Adachi Y Codon 54 polymorphism of the fatty acid binding protein gene and insulin resistance in the Japanese population Diabetes Med 1999 16 119 124 10.1046/j.1464-5491.1999.00034.x 10229304

[B29] Rissanen J Pihlajamaki J Heikkinen S Kekalainen P Kuusisto J Laakso M The Ala54Thr polymorphism of the fatty acid binding protein 2 gene does not influence insulin sensitivity in Finnish nondiabetic and NIDDM subjects Diabetes 1997 46 711 712 10.2337/diab.46.4.711 9075815

[B30] Chiu KC Chuang LM Yoon C The A54T polymorphism at the intestinal fatty acid binding protein 2 is associated with insulin resistance in glucose tolerant Caucasians BMC Genet 2001 2 7 11299043 10.1186/1471-2156-2-7 PMC31346

[B31] Weiss EP Brown MD Shuldiner AR Hagberg JM Fatty acid binding protein-2 gene variants and insulin resistance: gene and gene-environment interaction effects Physiol Genom 2002 10 145 157 10.1152/physiolgenomics.00070.2001 12209017

